# Current advances of *Dendrobium officinale* polysaccharides in dermatology: a literature review

**DOI:** 10.1080/13880209.2020.1787470

**Published:** 2020-07-11

**Authors:** Linghong Guo, Jinxin Qi, Dan Du, Yin Liu, Xian Jiang

**Affiliations:** a Department of Dermatology, West China Hospital, Sichuan University, Chengdu, Sichuan, China; b Department of Pharmacology, West China School of Basic Sciences & Forensic Medicine, Animal Research Institute, Sichuan University, Chengdu, Sichuan, China; c Department of Dermatology, The First People’s Hospital of Zigong, Zigong, Sichuan, China; d Department of Basic Medical Sciences, Sichuan Vocational College of Health and Rehabilitation, Zigong, Sichuan, China; e Department of Anesthesiology, School of Medicine, Sichuan Cancer Hospital & Institute, Sichuan Cancer Center, University of Electronic Science and Technology of China, Chengdu, Sichuan, China

**Keywords:** Natural products, cosmetics

## Abstract

**Context:**

*Dendrobium officinale* Kimura et Migo (Orchidaceae) is a naturally occurring precious traditional Chinese medicine (TCM) originally used in treating yin-deficiency diseases. The main active substances of *Dendrobium officinale* are polysaccharides (DOP). Recent findings highlighted the potential of DOP as a promising natural material for medical use with a diversity of pharmaceutical effects.

**Objective:**

In this review, we provide a systematic discussion of the current development and potential pharmacological effects of *Dendrobium officinale* polysaccharides in dermatology.

**Methods:**

English and Chinese literature from 1987 to 2019 indexed in databases including PubMed, PubMed Central, Web of Science, ISI, Scopus and CNKI (Chinese) was used. *Dendrobium officinale, Dendrobium officinale* polysaccharides, phytochemistry, chemical constituents, biological activities, and pharmacological activities were used as the key words.

**Results:**

*Dendrobium officinale* polysaccharides have been found to possess hair growth promoting, skin moisturising and antioxidant effects, which are highly valued by doctors and cosmetic engineers. We highlighted advances in moisturising and antioxidant properties from *in vivo* and *in vitro* studies. *Dendrobium officinale* polysaccharides exhibited strong antioxidant effects by decreasing free radicals, enhancing antioxidant system, inhibiting nuclear factor-kappa B and down-regulating inflammatory response.

**Conclusions:**

Our review is a foundation to inspire further research to facilitate the application of *Dendrobium officinale* polysaccharides in dermatology and promote active research of the use of TCM in dermatology.

## Introduction

*Dendrobium officinale* Kimura et Migo, a rare perennial herb, is the second largest genus of Orchidaceae (Jin and Huang 2015). It has a broad geographical distribution including India, Australia, Japan, and the United States, and it is widely spread in China (Lee et al. 2018). *Dendrobium officinale* is traditionally used as an ingredient in food or tea, since people believed it has an outstanding effect in balancing Yin and Yang (Teixeira da Silva and Ng 2017). In addition, studies have reported the combination of *D. officinale* and other tonic Chinese herb such as *Panax quinquefolii radix* (American Ginseng, Araliaceae) could enhance humoral immunity and cell-mediated immunity in mice (Tang et al. 2017). Considered to have the best medical properties among the *Dendrobium* species, *D. officinale* is a valuable and national key protected traditional Chinese medicine (TCM) (Jin and Huang 2015). Previous studies showed prominent benefit of using *D. officinale* for the treatment of atrophic gastritis, low-grade fever and mouth ulcers (Huang et al. 2016). Modern medicine has manifested a diversity of therapeutic effects of *D. officinale* encompassing anticancer, antiangiogenic, antiinflammatory, antioxidant, antidiabetic, immunoenhancing, hepatoprotective, antifungal, antibacterial, antiviral among other benefits (Teixeira da Silva et al. 2015; He et al. 2016; Luo et al. 2016). Recent research has confirmed that the major bioactive chemical compounds of *D. officinale* are polysaccharides possessing moisturising, antioxidant, anti-aging and hair growth promoting effects, which was highly valued by dermatologists and cosmetic engineers (Chen et al. 2014; Zhao et al. 2014; Teixeira da Silva et al. 2015).

The aim of this review is to integrate both *in vivo* and *in vitro* experimental research with available data on the current development and potential biological and pharmacological activity of *D. officinale* polysaccharides (DOP) in dermatology with special emphasis on the application in cosmetics. In addition, we hope our review of the TCM and ethnopharmacology could inspire more modern research to further investigate the TCM theory and develop the application of TCM in dermatology.

## DOP-major bioactive components of *D. officinale*

More than 190 compounds from *D. officinale* were isolated, mainly encompassing polysaccharides, amino acids, and other compounds (Lam et al. 2015). Among them, polysaccharides are the main active ingredients of *D. officinale* (Lo et al. 2004). Polysaccharides are complex carbohydrate polymers that are linked by a number of monosaccharide units via glycosidic bonds (Pan et al. 2014). Generally, three methods are adopted to isolate crude polysaccharides from *D. officinale*. Water extraction by alcohol sedimentation is the most common method (Chen et al. 2016; Tang et al. 2017). In addition, biological extraction using enzyme and physical extraction by ultrasonic wave and microwave are also feasible (Tang et al. 2017). After the extraction process, ion-exchange chromatography and gel filtration chromatography are usually used for purification (Chen et al. 2016; Ma et al. 2018). Notably, the polysaccharide content was highly associated with the production origin, growing years, harvest season, storage time, processing method, and different part of *D. officinale* (Teixeira da Silva et al. 2016). The total polysaccharide and mannose content are quantitative indicators of *D. officinale*. The Chinese Pharmacopoeia (2015) stipulates that the content of polysaccharides in *D. officinale* is more than 25%, and the content of mannose is about 13–38% (Hu et al. 2018). Previous studies have shown that the content of polysaccharides and mannose in only biennial *D. officinale* stems meets pharmacopoeial standards (Hu et al. 2018). The biennial and triennial *D. officinale* stems have much higher polysaccharide content than the leaves. In addition, researchers compared the effects of traditional hot water extraction, enzymatic extraction, and ultrasonic extraction on the polysaccharide content of *D. officinale* (Tan et al. 2019). It was found that the purity of DOP obtained by ultrasonic extraction was higher than that of enzymatic extraction and hot water extraction.

In recent years, there have been many reports on structural characteristics of *D. officinale* polysaccharides, mainly focussing on the molecular weight determination and monosaccharide composition analysis of crude polysaccharides. Presently, it has been found that *D. officinale* polysaccharide is mainly composed of mannose and glucose, and is rich in *O-*acetyl groups (Pan et al. 2014; Huang et al. 2015b). Previous researchers isolated three types of *D. officinale* polysaccharides, and measured their molecular weights to be 1000, 500, and 120 kDa, respectively, and determined that they were a class of *O-*acetyl*-*glucomannan (Wang et al. 1988). In another study, a heteropolysaccharide from an aqueous extract of *D. officinale* by anion exchange chromatography and gel permeation chromatography was obtained and identified as 2*-O-*acetylglucomannan (Luo et al. 2016). In addition, through Gas Chromatography-Mass Spectrometer (GC-MS), the monosaccharide components in *D. officinale* polysaccharides were identified to mainly include arabinose, galactose, glucose and mannose (Pan et al. 2014). Similarly, a low molecular weight polysaccharide extracted from the stem of *D. officinale* was found to be mainly composed of mannose and glucose in a molecular ratio of 4.5:1 (Xie et al. 2016). Noticeably, studies have demonstrated that the pharmacological activities of DOP are strongly related to the content and composition of their monosaccharides (Huang et al. 2016). Recently, researchers have conducted more in-depth analysis of structural characteristics of *D. officinale* polysaccharides. Gao et al. (2018) identified a homogeneous *D. officinale* polysaccharide from fresh *Dendrobium officinale* and found that DOP was a glucomannan with relatively large molecular weight and rich in *O-*acetyl group structure. The molar ratio of mannose residues to glucose residues was 4.9: 1. In addition, their results also showed that the content of sugar residues linked to multiple sites in DOP was not high. Therefore, DOP is a long-chain acetylated glucomannan with 1,4-linkage as the main branch. Acetylation mainly occurs at O-2 and O-3 positions, and only a few mannose residues are replaced by *O*-acetyl groups at O-2 and O-3 positions.

Numerous studies have focussed on the therapeutic functions of DOP, mainly for gastrointestinal diseases, hepatic diseases, diabetes, Sjogren's syndrome, and other internal diseases (Teixeira da Silva and Ng 2017). By contrast, biological and pharmacological effects of DOP on dermatology are relatively novel research topics.

## Antibacterial effect of DOP

It has been found that *Dendrobium officinale* polysaccharides have significant inhibitory effects on *Staphylococcus aureus*, *Bacillus subtilis*, *Candida albicans*, *Escherichia coli* and *Pneumococcus* (Zhang et al. 2012; Wang et al. 2016; Zhou et al. 2017). Among them, *Staphylococcus aureus* is the most common Gram-positive pathogen in dermatology (Wei et al. 2016). Common infectious skin diseases caused by *Staphylococcus aureus* include pustules, folliculitis, scabies, carbuncles, and staphylococcal scald skin syndrome (Wei et al. 2016). In addition, *Candida albicans* infection can cause dermatitis (Liu et al. 2014). Therefore, the antibacterial effect of DOP provides a basis for the research and development of natural antibacterial drugs. Subsequent studies should further validate its antimicrobial effects in skin disease models.

## Current use of DOP in hair growth promoting

As a hair disease, alopecia has a great influence on the mental and psychological health of patients (Rajoo et al. 2019). Currently, the prevention and treatment of alopecia mainly includes surgical treatment and/or drug treatment. As an invasive treatment, surgical treatment might cause complications such as bleeding, infection, swelling, hypertrophic scar, epidermoid cyst, etc. (Callender et al. 2014). Drug therapy prevents hair loss by promoting hair growth (Falto-Aizpurua et al. 2014). The commonly used drugs are minoxidil, finasteride, RU58841, cyclosporine A (CyA) and FK506, but they all have certain problems such as adverse effects and limited response (Kassira et al. 2017; Strazzulla et al. 2018). Therefore, the development of drugs with high efficiency, clear mechanism of action and mild or no side effects for prevention and treatment of alopecia has important social and economic significance.

Recently, Chen et al. (2014) performed an experimental study in which 30 C57BL6J mice were randomly divided into three groups and treated with *D. officinale* polysaccharides (5.0 g/L, minoxidil tincture and ultrapure water for 21 days, respectively. They found the average hair growth score and average quality of C57BL/6J mice in the DOP group were significantly better than those in the control groups. In addition, the survival rate of HaCaT cells and the expression level of vascular endothelial growth factor (VEGF) mRNA in the DOP group were significantly improved compared with those in the control groups. Studies have confirmed that hair follicles in the growing stage presented high vascularization, which was related to the expression of VEGF (Kozlowska et al. 1998; Hacker et al. 2010). Another study showed that VEGF could promote hair growth and thickened hair by inducing the formation of blood vessels around hair follicles (Yano et al. 2001). In addition, HaCaT cells were reported to possess the ability of synthesizing and secreting VEGF (Rho et al. 2005). In the experiment of Chen et al. (2014), the proliferation of HaCaT cells were found to be increased. Therefore, it was speculated that DOP might mediate the regeneration of hair follicle vessels and improve microcirculation by up-regulating the expression of VEGF mRNA, thereby promoting hair growth.

## Potential effects of DOP in cosmetics

A distinctive feature of TCM is the individualized treatment based on identifying the ‘syndrome’. In the methodology that can be used to identify different ‘syndrome’, the Eight Guiding Principles (EGP) divide the disease or syndrome into eight sub-patterns, and group them into opposite pairs: Yin-Yang; Exterior-Interior; Cold-Heat; and Deficiency-Excess (Lee et al. 2007). Health is believed to be a balanced state of Yin and Yang and disease is the consequence of imbalance of these two forces. In TCM, Yin refers to general body fluids including blood, bodily fluids ans Jing (essence). ‘Yin Deficiency’ is defined as a pathological state associated with Yin-related dysfunction, the syndrome of which is usually manifested by the diminishment in calming, moistening and Yang heat regulation. Consequently, the weakening of Yin’s control over Yang leads to hyperactivity of Yang (Yang flourishing) (Hu et al. 2019). According to the theory of the traditional Chinese medicine, *D. officinale* is the best product for nourishing Yin, which means it is able to treat Yin Deficiency related syndromes (Teixeira da Silva and Ng 2017). Acne, pustule and some other dermatologic disorders were considered to be caused by Yin deficiency and Yang flourishing in TCM (Li et al. 2015). Chinese people have believed the application of *D. officinale* can effectively solve the above problems. *D. officinale* is rich in polysaccharides, which can be absorbed not only by oral administration, but also by skin (Pan et al. 2014). Therefore, the active substance and functional component DOP in *D. officinale* can be extracted and possessed into cosmetic products, which might play a good moisturizing, antiaging, nurturing, and inflammation inhibiting effect.

### Skin-moisturizing effect

Skin moisturizing is one of the basic functions of skin care cosmetics. Due to the increase of age and the influence of the external environment, the moisturizing structure of the skin will be damaged, and the water content between the cells of the skin will be reduced, which will lead to the tight arrangement of the cells and sclerosis of collagen (Zulkowski 2017). When the water content in the stratum corneum drops to less than 10%, the skin will appear dry, lose elasticity, wrinkle and accelerate skin aging (Choi 2019). Currently, hyaluronic acid is widely used in cosmetics as a moisturizer, but it has some disadvantages in the process of application (Salwowska et al. 2016). Hyaluronic acid is degraded into small molecules of hyaluronic acid by enzymes, which might cause allergies (Signorini et al. 2016). On the other hand, due to the high molecular weight of hyaluronic acid, there are certain difficulties in the application of the formulation (Schelke et al. 2019). Therefore, people are gradually focussing on finding alternatives from natural plants.

Notably, the active component polysaccharides in *Dendrobium officinale* have excellent moisturizing effect (Lam et al. 2015). The polysaccharide content of *D. officinale* is significantly higher than that of other *Dendrobium* species (Lee et al. 2018). The biological activity and physicochemical properties of polysaccharides provide a basis for their good moisturizing properties. Polysaccharide has good film-forming performance, which can form a uniform thin film on the skin surface to reduce the evaporation of water on the skin surface (Vijayendra and Shamala 2014). Thus, the water is diffused from the basal tissue to the stratum corneum and induce further hydration of the keratin layer (Gao et al. 2019). In this process, the skin moisture is preserved. In addition, hydroxyl, carboxyl, and other polar groups in the polysaccharide molecule can form hydrogen bonds with water molecules to bind a large amount of water (Li et al. 2017). Meanwhile, the molecular chains of the polysaccharides are also intertwined into a network, which plays a strong role in water retention (Sebti and Coma 2002). Therefore, the perfect combination of good film forming property and high water-absorption of the polysaccharide provide a good moisturizing effect to the skin. Some studies have experimentally verified the moisturizing properties of DOP. Chen et al. (2015) obtained *D. officinale* polysaccharide extract by water extraction and studied its moisturizing performance. The results manifested that the extract of *D. officinale* showed the same moisturizing effect as sodium hyaluronate at 2 h and 4 h, and the moisturizing rate was close to 50% of sodium hyaluronate at 6 h. The extracted polysaccharide could significantly resist the damage of epidermis cells caused by drying and increase the cell vitality in a concentration-dependent manner.

In summary, the bioactive polysaccharides in *D. officinale* might serve as moisturising factors and have good value in the application of cosmetics.

### Antiaging effect by alleviating oxidative stress

#### Oxidative stress and aging of human skin

Oxygen is the most important energy to sustain life (Natarajan et al. 2014; Kammeyer and Luiten 2015; Lephart 2016). In the process of human metabolism, there has to be enough oxygen, and various nutrients need to be combined with oxygen to complete the physiological oxidation process and generate energy (Lephart 2016). However, oxygen can also cause oxidative damage to life. Because of their special electron arrangement, oxygen molecules (O_2_) are extremely easy to form free radicals during the process of metabolism, which are collectively known as reactive oxygen species (ROS), including superoxide anion (O_2_^-^), hydroxyl radical (OH) and hydrogen peroxide (H_2_O_2_) (Maes et al. 2011; Luderer 2014; Rinnerthaler et al. 2015). Normally, our body has two major defense systems against ROS (Lei et al. 2016). One is the free radical detoxifying enzymes, briefly composed of superoxide dismutase (SOD), catalase (CAT) and glutathione peroxidase (GSH-Px) (Ahmadinejad et al. 2017). SOD is an antioxidant metalloenzyme that can catalyse the disproportionation of superoxide anion radicals (O_2_^-^,) to form oxygen or H_2_O_2_ (Bresciani et al. 2015). CAT is another important enzyme that converts H_2_O_2_ to water (H_2_O) and O_2_, thus completing the action of SOD (Glorieux et al. 2015). GSH-Px is similar to CAT and convert H_2_O_2_ into H_2_O and O_2_ (Cardoso et al. 2017). The other defense mechanism is the antioxidant molecules. Antioxidants are capable of scavenging or inhibiting the production of free radicals, blocking the spread of free radical chain reaction, and terminating the process of free radical reactions (Rizzo et al. 2010). Uric acid and glutathione are common endogenous antioxidants (Pisoschi and Pop 2015). Many antioxidants are obtained from diet or other exogenous sources (Lei et al. 2016). In recent years, antioxidants such as phenolic compounds and polysaccharides are found abundant in many plants (Szymanska et al. 2016). Many of these antioxidants are integrated into the epidermis and dermis of the human skin (Lephart 2016). Notably, both free radical defensing enzyme and antioxidants are essential for maintaining the dynamic balance of the production and elimination of free radicals *in vivo* (Maes et al. 2011). Once this balance is broken, there will be excessive production of ROS, resulting in oxidative stress (Pisoschi and Pop 2015). Oxidative stress can have deleterious effects on biological molecules and structures, including damage to DNA, RNA, proteins, lipids and membranes (Bar-Or et al. 2015). Therefore, oxidative stress is involved in many disorders of major organ systems such as skin, cardiovascular, respiratory, immune and skeletal (Hybertson et al. 2011; Gorrini et al. 2013; Kirkham and Barnes 2013).

**Figure 1.**
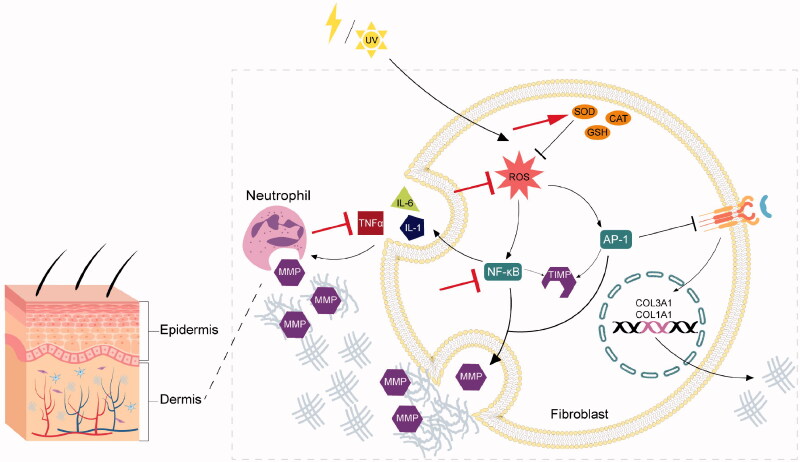
Skin aging via oxidative mechanism and the antiaging effect of DOP. The internal cause of skin aging occurs in the dermis, which is a dense network of fibres and elastic tissue. ROS are generated by UV radiation and other factors. In direct or indirect ways, ROS can activate various intracellular kinases leading to the production of AP-1 and NF-κB. Activated AP-1 and NF-κB regulate the transcription of matrix MMPs, which can degrade collagen and elastin. In addition, NF-κB is a major activator of inflammatory cellular infiltration by inducing the production of pro-inflammatory cytokines TNF-α, IL-1 and IL-6, which stimulates further production of MMPs. ROS-induced AP-1 also down-regulates the TGF-β receptor and impairs subsequent cascade and pathways, which cause the reduction of collagen type I and III. Ultimately, the collagen and elastin level in dermal layer is reduced and therefore accelerating skin aging. DOP can achieve antiaging functions by defensing oxidative damage in the following aspects, including enhancing antioxidant enzyme system (red arrow), decreasing ROS, inhibiting NF-κB and inhibiting inflammatory response (red T icon). DOP: Polysaccharides of *D. officinale*; ROS: Reactive Oxygen Species; AP-1: Activator Protein-1; NF-κB: Nuclear Factor-kappa B; MMPs: Matrix Metalloproteinase; TNF-α: Tumour Necrosis Factor-α; IL-1β: Interleukin-1beta; TGF-β: Tumour Growth Factor-β.

Researches revealed that the underlying mechanism of skin aging were associated with ROS generation and subsequent oxidative stress (Imokawa and Ishida 2015). ROS are generated by the mitochondria, UV radiation and other extrinsic factors (Pattison and Davies 2006). In direct or indirect ways, ROS can activate various intracellular kinases including MAPK, AKT and ERK, leading to the production of activator protein-1 (AP-1) and nuclear factor-kappa B (NF-κB) (Fisher et al. 1998). Activated AP-1 and NF-κB regulate the transcription of matrix metalloproteinases (MMPs) (Whitmarsh and Davis 1996). Four members of MMPs encompassing MMP1, MMP2, MMP3 and MMP9 working together can fully degrade collagen (Ashworth et al. 1999). Notably, MMPs are the members of the elastase family with the capacity of degrading elastin and decreasing elastin levels (Birkedal-Hansen 1987). At the same time, activated AP-1 and NF-κB can also increase the expression of MMP tissue inhibitor (TIMP) (Whitmarsh and Davis 1996). Nonetheless, the MMP activation still occupies comparative predominance over the activity of TIMP, resulting in the degradation of collagen, elastin, fibrillar and some other dermal extracellular matrix (Birkedal-Hansen 1987). In addition, NF-κB is a major activator of inflammatory cellular infiltration by inducing the production of pro-inflammatory cytokines TNF-α, interleukin (IL)-1, IL-6 and VEGF, which stimulates further production of MMPs (Huang and Xin 2018). ROS-induced AP-1 also decreases the biosynthesis of collagen (Whitmarsh and Davis 1996). Since AP-1 downregulates the transforming growth factor-β receptor and impairs subsequent cascade and pathways, which cause the reduction of collagen type I and III (Yu et al. 2013). Ultimately, the collagen and elastin level in dermal layer is reduced and therefore accelerating skin aging. Figure 1 summarised the mechanism of oxidative stress leading to skin aging.

#### DOP’s antioxidant effect on human skin

Studies have indicated that many components of *D. officinale* including polysaccharides, flavonoid and vitamin C (VC) could alleviate oxidative stress in cascade reaction, which might be beneficial for fighting skin aging (Huang et al. 2016; Zeng et al. 2017; Mai et al. 2019). Among them, polysaccharides of *D. officinale* are the most important and multi-directional antiaging ingredients.

Researchers found DOP and its two isolated fractions (DOPA-1 and DOPA-2) exerted a cytoprotective effect on H_2_O_2_-treated RAW 264.7 macrophages (Huang et al. 2016). Specifically, their results revealed that pre-treatment with DOP, DOPA-1 and DOPA-2 markedly promoted cell viability, suppressed apoptosis and ameliorated oxidative lesions. In 2017 Zeng et al. first reported the gastroprotective effect of DOP by inhibiting oxidative stress-triggered apoptosis. In the *in vitro* study, the destructive oxidative damage induced by H_2_O_2_ in HFE145 cells were reversed by the addition of DOP in advance. In animal experiments (Mai et al. 2019), researchers also discovered the reparative effect of *D. officinale* protocorms against oxidative damages caused by UV radiation. They compared the effect of *D. officinale* protocorms and matrixyl on UV radiation induced photodamage in hairless mice. Their results revealed that *D. officinale* protocorms protected the skin from dryness and effectively reduced erythema by enhancing the antioxidant systems.

Regarding the specific mechanism of antioxidation of *D. officinale*, we have summarised the findings of various investigators below.

#### DOP decreases free radicals (ROS)

Accumulating evidence showed that *D. officinale* polysaccharides exhibited valuable high free radicals scavenging activity (Luo et al. 2016; Xing et al. 2018). In a recent report, a water-soluble polysaccharide obtained from *D. officinale* has been proved to possess excellent scavenging activity of hydroxyl radical and 1,1-diphenyl-2-picrylhydrazyl (DPPH) (Luo et al. 2016). Xing et al. (2018) adopted the ethanol extract method to obtain four purified fractions of DOP (DOP-40, DOP-50, DOP-60 and DOP-70) and found that DOP-40, DOP-60 and DOP-70 exhibited high scavenging activity of O_2_^-^, DPPH and hydroxyl radicals. Among them, DOP-70 demonstrated the strongest antioxidant ability. The free radical scavenging ability of DOP fractions in their study showed concentration dependence.

#### DOP enhances antioxidant systems

Results of recent *in vivo* and *in vitro* experiments demonstrated that DOP could enhance the effects of antioxidant defense system to achieve antioxidant capability (Pan et al. 2014; Huang et al. 2015b; Luo et al. 2016; Zhang et al. 2016; Zhao et al. 2017). Huang et al. (2015b) investigated the antioxidant activities of *D. officinale* and its two polysaccharide fractions in cyclophosphamide (CTX) induced immunosuppressed mice. Accumulating reports indicated that CTX exposure stimulated the production of intracellular ROS and subsequent oxidative stress during the immunosuppression process (Jeelani et al. 2017). All sample tests in their study showed the protective effect of *D. officinale* from oxidative injuries by increasing the activities of SOD, CAT and GSH-Px in the serum, liver and thymus. In addition, they also found the decrease of malondialdehyde (MDA) content in *D. officinale* treated animals. MDA is known to act as an oxidative stress marker (Draper and Hadley 1990). Similarly, in another study which adopted alloxan to trigger oxidative stress, all perturbations caused by oxidative stress were significantly restored by the administration of DOP via improving the activities of CAT, SOD and GAH and decreasing the level of MDA in the liver and kidney (Pan et al. 2014). In 2017, researchers found that pre-treatment with DOP could attenuate H_2_O_2_-induced oxidative injuries in H9c2 cells (Zhao et al. 2017). Examination of cultured supernatant manifested an increased SOD activity and decreased MDA level in the DOP-treated group. Metal chelation activity represents one of the antioxidant mechanisms since it reduces the metal concentration required for lipid peroxidation (Canabady-Rochelle et al. 2018). Studies also revealed that DOP possessed valuable high metal chelating activity (Luo et al. 2016). In addition, one study reported that *D. officinale* extracts (DOE) attenuated diabetic cardiomyopathy via the inhibition of oxidative stress (Zhang et al. 2016). More specifically, results in this study showed that pre-addition of DOE significantly increased the production of T-SOD and inhibited the activities of MDA.

#### DOP inhibits NF-κB

NF-κB is a pro-inflammatory transcription factor, which could be found in almost all animal cells (Hayden and Ghosh 2008). NF-κB plays a key role in the oxidative stress mechanism and the pathophysiology of inflammatory diseases (Lephart 2016). We previously mentioned that NF-κB could increase the level of MMP in different ways and therefore inducing the degradation of collagen and elastin. Activated NF-κB might be associated with the generation of haem oxygenase-1, which elevates the level of free iron and induces further ROS production via the Fenton reaction (Rinnerthaler et al. 2015). Therefore, NF-κB is highly related to the oxidative injuries and involved in the mechanism of skin aging.

Several investigators have explored the inhibitory effect of DOP on NF-κB. Zeng et al. (2017) demonstrated that DOP could protect cells against oxidative injuries-induced apoptosis via inhibition of NF-κB activation. Their results showed that pre-treatment of DOP dramatically reduced the NF-κBp65/p-NF-κBp65 expression induced by H_2_O_2_. In addition, Xiang et al. (2013) found that the pre-addition of DOP inhibited the ROS generation induced by TNF-α. At the same time, they observed the pre-treatment of DOP suppressed the translocation of NF-κB to nuclei. These results indicated that DOP might be beneficial for antiaging via the inhibition of NF-κB.

#### DOP inhibits inflammatory response

During the last two decades, extensive research has revealed that inflammatory response and oxidative stress mediate each other, indicating that inflammation might lead to skin damage via oxidative injuries (Hybertson et al. 2011). It is also known that various pro-inflammatory factors are increased with UV exposure (Bald et al. 2014). As we discussed above, several pro-inflammatory biomarkers including TNF-α, IL-1, IL-6 could stimulate the production of MMP and therefore causing further degradation of collagen and elastin and accelerating skin aging. Previous studies also confirmed that TNF-α could promote the generation of ROS, which was the major cause of skin aging (Fischer and Maier 2015).

It has been shown that DOP could suppress inflammatory responses. Lin et al. (2011) found that DOP reduced the expression of pro-inflammatory cytokines including IL-1β, IL-6 and TNF. It is worth noting that they also discovered decreased activity of MMP-9 in the DOP-treated group, which suggested that less degradation of the extracellular matrix. In addition, Zhang et al. (2016) investigated the potential role of DOE in diabetic cardiomyopathy and found that DOE downregulated the activities of TNF-α and IL-1β, indicating that DOE might have protective potential for human against oxidative damage via inhibition of several pro-inflammatory factors.

**Figure 2.**
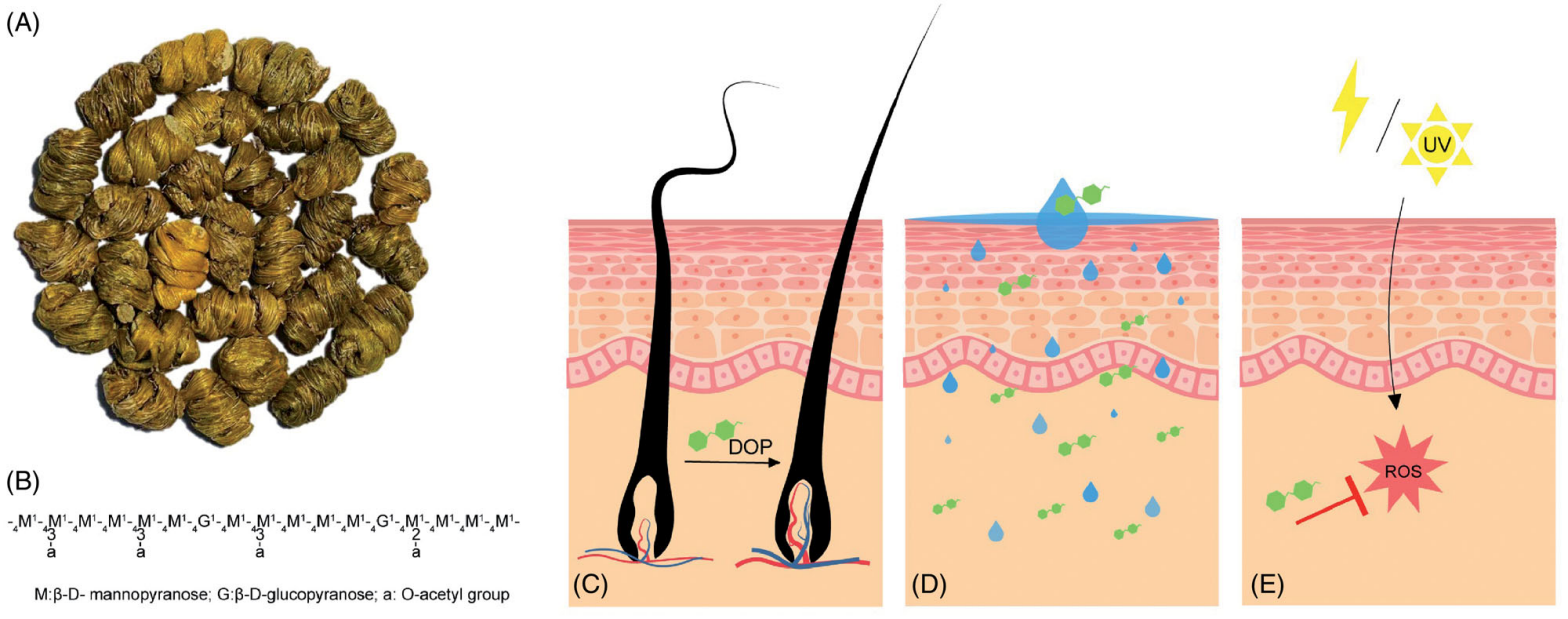
Pharmacological effects of DOP in dermatology. (A) *Dendrobium officinale.* (B) Dendronan. Primary structure of DOP is mainly composed of *O*-acetylated sugar residues. (C) Hair growth promoting effect of DOP. (D) Skin-moisturising effect of DOP. (E) Antioxidant effect of DOP. DOP: Polysaccharides of *D. officinale*.

The potential effect and corresponding active component of *D. officinale* in cosmetics were summarised in Table 1 and the potential pharmacological effects of DOP in dermatology were shown in Figure 2.

**Table 1.** The potential effect of *Dendrobium officinale* in cosmetics.

Potential effect	Active component	*In vivo*/*vitro*	Observations	Relevant mechanism	Reference
Skin-moisturizing effect	DOP	*In vitro*	Showed the same moisturising effect as sodium hyaluronate at 2 h and 4 h, and the moisturising rate close to 50% of sodium hyaluronate at 6 h Significantly resisted the damage of epidermis cells caused by drying and increased the cell vitality in a concentration-dependent manner	Good film-forming property and high water-absorption ability	(Chen et al. 2015)
Antioxidant effect	DOP, DOPA-1 and DOPA-2	*In vitro*	Showed a cytoprotective effect on H_2_O_2_-treated RAW 264.7 macrophages	Promoted cell viability, suppressed apoptosis and ameliorated oxidative lesions	(Huang et al. 2016)
Antioxidant effect	DOP	*In vitro*	Exhibited gastroprotective effect against H_2_O_2_-induced oxidative stress	Improved age nuclei morphology changes and remarkably decreased the number of apoptotic cells	(Zeng et al. 2017)
Antioxidant effect	*D. Officinalis* protocorms	*In vitro*	Showed reparative effect against oxidative damages caused by UV radiation	Protected the skin from dryness and effectively reduced erythema by enhancing the antioxidant systems	(Mai et al. 2019)
Antioxidant effect	DOP	*In vitro*	Showed excellent scavenging activity of hydroxyl radical, DPPH radical	Decreased free radicals (ROS)	(Luo et al. 2016)
Antioxidant effect	DOP-40, DOP-60 and DOP-70	*In vitro*	Exhibited high scavenging activity of O_2_^-^, DPPH and hydroxyl radicals	Decreased free radicals (ROS)	(Xing et al. 2018)
Antioxidant effect	*D. Officinalis* and two DOP fractions	*In vivo*	Showed protective effect against oxidative injuries by increasing the activities of SOD, CAT and GSH-Px in the serum, liver and thymus and decreasing MDA content	Enhanced antioxidant system	(Huang et al. 2015b)
Antioxidant effect	DOP	*In vivo*	Restored all perturbations caused by oxidative stress via improving the activities of CAT and SOD as well as the contents of GSH and decreasing the level of MDA in the liver and kidney	Enhanced antioxidant system	(Pan et al. 2014)
Antioxidant effect	DOP	*In vitro*	Attenuated H_2_O_2_-induced oxidative injuries in H9c2 cells by increasing SOD activity and decreasing MDA level	Enhanced antioxidant system	(Zhao et al. 2017)
Antioxidant effect	DOP	*In vitro*	Possessed high metal chelating activity	Enhanced antioxidant system	(Luo et al. 2016)
Antioxidant effect	DOE	*In vivo*	Attenuated diabetic cardiomyopathy by increasing the production of T-SOD and inhibiting the activities of MDA	Enhanced antioxidant system	(Zhang et al. 2016)
Antioxidant effect	DOP	*In vitro*	Protected cells against oxidative injuries-induced apoptosis via inhibition of NF-κBp65/p-NF-κBp65 expression induced by H_2_O_2_	Inhibited NF-κB	(Zeng et al. 2017)
Antioxidant effect	DOP	*In vitro*	Suppressed the translocation of NF-κB to nuclei	Inhibited NF-κB	(Xiang et al. 2013)
Antioxidant effect	DOE	*In vivo*	Alleviated diabetic cardiomyopathy and down-regulated the activities of TNF-a and IL-1β	Inhibited inflammatory response	(Zhang et al. 2016)
Antioxidant effect	DOP	*In vivo*	Reduced the expression of pro-inflammatory cytokines including IL-1β, IL-6 and TNF	Inhibited inflammatory response	(Lin et al. 2011)

DOP: polysaccharides of *D. officinale*; N/A: not applicable; DOE: *D. officinale* extracts; ROS: reactive oxygen species; SOD: superoxide dismutase; CAT: catalase; GSH-Px: glutathione peroxidase; MDA: malondialdehyde; NF-κB: nuclear factor-kappa B; TNF-α: tumour necrosis factor-α; IL-1β: interleukin-1beta.

## DOP’s bioactivity is related to its structural characteristics

It is known that the major active substance of *D. officinale* is the polysaccharide. The bioactivity of DOP is closely related to its chemical composition, structural features, molecular weight, etc. (Methacanon et al. 2005). Some studies have indicated that polysaccharides with high mannose content have excellent bioactivities. Meng et al. (2015) demonstrated a statistically significant correlations between the antioxidant capability of polysaccharide and the monosaccharide composition (mannose, *r* = 0.942). In another research, two purified fractions of DOP with high mannose content were found to possess strong ability to ameliorate oxidative lesions (Huang et al. 2016). In addition, studies on antioxidant activity of polysaccharides, sulphated polysaccharide and acidic polysaccharide are frequently reported. Researchers have showed that the scavenging superoxide radical capacity was positively influenced by the sulphate content of polysaccharide (Wang et al. 2008). Another study indicated that the most potent antioxidant ability of sulphated polysaccharide was gained from the esterified carboxyl residue (Hu et al. 2001). Acidic polysaccharide with the content of uronic acid was also proved to be a major reason for its antioxidant activity (Chen et al. 2004). In addition, Chen et al. (2004) compared the ROS scavenging abilities of tea polysaccharide conjugates with various uronic acid contents. They discovered that high content of uronic acids was related to stronger ROS scavenging activities. Interestingly, investigators found that DOP mainly with neutral sugars also exhibited excellent protective effects on oxidative damage (Huang et al. 2015a). Further research revealed that they contained 1, 4-linked β-d-Manp and *O*-acetyl groups (Yang et al. 2014). The existence of 1, 4-linked β-d-Manp and *O*-acetyl groups was reported in the polysaccharides of many *Dendrobium* species including *Dendrobium huoshanense* C. Z. Tang et S. J. Cheng, *Dendrobium tosaense* Makino and *D. officinale*, and was found to be the possible main structure contributing to its antioxidant activity (Huang et al. 2016). Moreover, molecular weights of polysaccharides were demonstrated to be positively related to their bioactivities (Wang et al. 2013). Specifically, studies indicated that polysaccharides with molecular weight greater than 100 kDa exhibited more excellent bioactivity.

## The toxicology of *D. officinale*

Several studies have been performed to evaluate the safety of *D. officinale*. Researchers have conducted acute oral toxicity test, Ames test, micronucleus test of bone marrow and sperm malformation test in mice treated with 15% decoction of *D. officinale* (Xu et al. 1998). Among them, Ames test was performed to reflect gene mutation and micronucleus test of bone marrow was designed to evaluate the chromosomal damage. Ames test, micronucleus test of bone marrow and sperm malformation test all belongs to genetic toxicity test. All test results were negative and the highest dose in acute oral toxicity test was up to 10 g/kg b.w., which was equivalent to 600 g for adults, suggesting that there is no mutagenic effect of *D. officinale* extract. The safety of *Dendrobium officinale* protocorms was also assessed by acute toxicity test, genetic toxicity test and 90-day feeding test in mice (Feng et al. 2014). The results revealed that *D. officinale* protocorms were without toxicity, genetic toxicity and mutagenicity within the scope of the test dose. Recently, the effect of *D. officinale* on pregnant rats and embryonic development of foetal rats were investigated in an animal experiment (Qin et al. 2019). The authors found that *D. officinale* had no obvious effect on pregnant rats and no deformity effect on foetal rats, but it could cause foetal rats to gain weight. However, there is little research on the skin toxicity of topical use of *D. officinale*. The safety and efficacy of long-term use of *D. officinale* is still uncertain. More in-depth research on the safety of *D. officinale* is urgently needed.

## Conclusions and future prospects

The clinical application of traditional Chinese medicine is rapidly expanding. Among them, the biological effect of DOP is one of the hotspots of research projects worldwide. However, there are few reports on the application of DOP in the field of dermatology. In this review, we have focussed on the current progress and the potential function of DOP in dermatology. DOP was shown to be a potentially promising anti-aging therapeutics due to its favourable moisturising and antioxidant effects. Specifically, DOP possessed strong antioxidant effects via various mechanisms including decreasing free radicals, enhancing antioxidant system, inhibiting nuclear factor-kappa B and down-regulating inflammatory response. However, these effects and underlying mechanisms still need to be further investigated and verified by additional *in vitro* and *in vivo* experiments of DOP. In addition, investigations about the relationships between polysaccharide structure and its function remains scant. Moreover, DOP has to be systematically tested for toxicity before it can be applied to cosmetics in direct contact with the skin. However, currently, there is no sufficient testing data about its safety.

With the increased awareness of the potential value of *D. officinale*, the demand for and price of *D. officinale* are also rising. As a consequence, the yield of *D. officinale* appears insufficient and the falsification and adulteration of *D. officinale* increases. Strengthening protection and expanding artificial cultivation are effective means for its sustainable development. At the same time, microscopic identification, DNA molecule marker technologies, pharmacognosy and other detecting methods have been developed to identify *D. officinale* from other confusable species. However, there is still a need to establish an effective quality control and evaluation system for *D. officinale*.

Overall, *Dendrobium officinale* polysaccharide is a potentially valuable and attractive natural material for the application in the field of dermatology, especially for its moisturising and antioxidant functions for skin care and cosmetics. More thorough studies are required to further assess the direct effect of *Dendrobium officinale* polysaccharides on skin, the structure-activity relationships, the safety and the quality control and evaluation of *Dendrobium officinale*.
